# The Bubbleverse: Women’s Experiences of Pelvic Floor Care and Their Journey Through Bubbles

**DOI:** 10.1007/s00192-026-06770-w

**Published:** 2026-06-16

**Authors:** Semra Sahin-Haglund, Thomas Torstensson, Jimmy Norén, Per-Olof Hedvall, Sahruh Turkmen

**Affiliations:** 1 Faculty of Engineering, Design Sciences, Certec, Lund University, Lund, Sweden; 2 Department of Public Health and Caring Sciences, Family Medicine and Preventive Medicine Section, Uppsala University, Uppsala, Sweden; 3 Department of Natural Sciences, Design and Sustainable Development, Mid Sweden University, Sundsvall, Sweden; 4 Department of Clinical Sciences, Section: Obstetrics and Gynecology, Umeå University, Umeå, Sweden

**Keywords:** Pelvic floor injury, Person-centred care, Postpartum, Qualitative study, Universal design

## Abstract

**Introduction:**

This study explores women’s experiences of pelvic floor (PF) care after childbirth, revealing significant insights into their physical and emotional journeys during the transformative period following birth in a Swedish tertiary hospital context. The focus is on women who sustained third- or fourth-degree PF injuries after vaginal birth, viewed through the perspective of universal design (UD).

**Methods:**

The study uses qualitative methods, observations, and interviews to illuminate challenges in PF care. Qualitative content analysis is used to identify themes emerging from participants’ narratives. Comics serve as a visual and multimodal method to depict the variety and sequencing of women’s narratives in multimodal ways.

**Results:**

The analysis identified two main themes. The first, the Bubbleverse, is a metaphor for women’s experiences across interconnected phases of birth and recovery, where PF injuries were often overshadowed by vaginal birth, newborn care, and future uncertainty. The second theme reflected women’s recommendations for healthcare, calling for timely, individualised communication, and structured support.

**Conclusions:**

The study concludes with a call to reconfigure the fragmented and underprioritised aftercare. Alternative care pathways tailored to individual needs are essential for more equitable PF care.

## Introduction

Giving birth is a profound life event. Attention shifts from the pregnancy to the newborn baby, both for parents and healthcare providers. However, the impact of childbirth on a woman’s body remains relatively under-discussed, despite its critical importance.

The birth plan letter is a Swedish practice [1] written by the pregnant woman, midwife, and sometimes the partner. It outlines the woman’s wishes and expectations for childbirth while acknowledging that not all of them may be fulfilled.

While plans for pregnancy, childbirth, and newborn care are standardised in Sweden, postpartum care for pelvic floor (PF) injuries varies between regions and lacks national standardisation. Responsibility for seeking care is often placed on the woman herself. Recent national guidelines emphasise the need to strengthen postpartum care for PF dysfunction and to address taboos surrounding symptoms such as urinary leakage (and, for some women, fecal leakage), while improving equitable access to care [2, 3].

The emotional and cognitive state following childbirth has been described through concepts such as the halo effect and maternity blues [4, 5]. The emotional intensity of birth, combined with the focus on the newborn and the immediate postpartum period, may obscure ongoing health challenges. Figurative descriptions such as being *in a bubble* or *on another planet* reflect altered states that may limit awareness or articulation of injury. These dynamics may contribute to delayed recognition of symptoms and the deprioritisation of PF care in the postpartum period. Comics-based research, an emerging multimodal approach that uses illustration as a tool for inquiry [6], may help visualise complex experiences and support the interpretation and communication of women’s narratives about PF injury.

Universal design (UD) emphasises equity, non-stigmatising approaches, and the inclusion of marginalised groups in development processes [7] supporting more holistic and inclusive postpartum care. A UD perspective, used alongside comics-based methods, may help illuminate the complexity of these experiences.

### Aim

This study aims to deepen the understanding of women’s experiences following grade three or four PF injuries through observations and interviews, using the UD framework to strengthen equity and quality of care.

### Research Questions

RQ1. What characterises the women’s experiences of the early postpartum period following a severe PF injury?

RQ2. How can UD be used as a critical perspective to explore the narratives of women with PF injuries?

## Theory

Suchman’s theory on Situated Action states that people’s actions can never be entirely anticipated and are continually evolving through unpredictable interaction with their surroundings [8]. Vaginal birth exemplifies this unpredictability, where expectations and written birth plans may prove inadequate in the face of unexpected events, such as a severe PF injury. The experience of childbirth, shaped by cultural, medical, and personal expectations, can reveal a gap between anticipated ideals and lived reality, particularly for women who sustain severe PF injuries and must manage the physical and emotional consequences. Suchman’s perspective emphasises that the fundamentals of action, whether deliberate or spontaneous, are situated within a specific context [8]. This study recognises that women’s experiences of PF injury can be understood through situated practices or interactions, as revealed through contextual observations and interviews.

Formerly, UD was described as a strategic approach aimed at designing environments, products, and services accessible to all people regardless of age, disability, or other factors [9]. A recent description of UD is more relevant as it addresses an attitude change in society, stressing democracy, equity, and citizenship, defined as a process rather than an outcome [7]. UD emphasises inclusivity through targeted interventions that prioritise vulnerable populations. This approach challenges minimal compliance requirements or isolated after-adaptations, instead embracing inclusivity as a holistic practice shaping design. In the context of PF care, applying UD ensures that postpartum recovery pathways consider the diverse physical, emotional, and social needs of women, particularly those often marginalised in healthcare.

In this article, UD serves as a guiding framework for examining how PF care after childbirth can be made more equitable and responsive to women’s needs. The framework encourages critical reflection on healthcare practices that may overlook the lived experiences of women with severe PF injuries by highlighting issues of equity, inclusion, and participation. In this study, the UD framework informed the study design, development of the interview guide, and interpretation of findings.

## Method

This study uses an exploratory qualitative design, combining observations and semi-structured interviews, to deepen the understanding of women’s experiences of PF injury and PF care.

### Participants and Recruitment Process

This study was conducted in a Swedish tertiary hospital, focusing on women who sustained third- or fourth-degree perineal tears after vaginal birth and attended routine postnatal follow-up visits, which, according to local clinical routines, take place between 8 and 12 weeks postpartum. Participants varied in personal birth and recovery experiences but shared the common context of severe PF injury. Interviews and observations were conducted in private hospital settings to capture in-depth, experiential narratives. Ten women were recruited between June 2024 and June 2025. Participants were purposively selected, had delivered healthy infants, and were accompanied by a partner during labour (Table 1). Participants who sustained perineal tears during childbirth received primary repair according to standard clinical practice immediately after birth. Despite this repair, some injuries can result in persistent PF dysfunction, which is why participants were attending postnatal follow-up and were eligible for inclusion in the study. Exclusion criteria included age under 18, current pregnancy, mental illness, and known or suspected pelvic or urinary tract infections.

**Table 1:** Participants’ characteristic

Participant	Age	Education	Parity	Mode of birth
P1	32	Higher education	Multipareous	NVB
P2	24	Higher education	Nullipareous	NVB
P3	29	High School education	Nullipareous	IVB
P4	35	Higher education	Multipareous	IVB
P5	40	Higher education	Nullipareous	NVB
P6	32	High School education	Nullipareous	NVB
P7	24	High School education	Nullipareous	NVB
P8	26	High School education	Nullipareous	NVB
P9	27	Higher education	Nullipareous	NVB
P10	24	Higher education	Multipareous	NVB

*P* Participant, *NVB* Normal vaginal birth, *IVB* Instrumental vaginal birth

All participants spoke fluent Swedish

Eligible patients were identified using ICD-10 codes O70.2 and O70.3, which correspond to third- and fourth-degree PF injuries. Obstetrical data were retrieved from the digital record system called *Obstetrix*. Inclusion and exclusion criteria were applied to ensure proper selection. Study invitations were sent along with routine follow-up appointment letters. Interested patients received oral and written information during their clinic visit, and written informed consent was obtained. Participants were informed of their rights, including the option to withdraw at any time, omit uncomfortable questions, and the assurance that their medical care would not be affected.

### Procedure

The participants were observed during a postpartum follow-up examination for their PF injury, conducted by a doctor, and interviewed by the researcher immediately afterwards. During vaginal examinations, participants were offered the option for the researcher to leave the room; nearly half accepted. The researcher’s position in the consultation room during observations was guided by healthcare staff and limited by room layout and furniture.

The interviews took place in a private room within the clinic. The layout of the interview room included a large conference table with chairs, with the interviewer opposite the participant, alongside the interview questions and the voice recorder on the table. The first author conducted the interviews, sometimes with the newborn present. All interviews were audio-recorded for transcription.

### Observations

Nonparticipant, semi-structured qualitative observations were used to explore the care context [10], combined with sketches. Observations focused on gathering insights without engaging in activities. For consenting participants, Biodesign’s Observation Documentation Guidelines were followed [11], capturing date, time, context, all attendants, specific facts, numbers, details, sensory impressions, specific words, phrases, timing of various steps of a process, procedure, interaction, and interaction with healthcare [11]. Observation results include researchers’ notes and sketches.

### Semi-structured Interviews

Semi-structured interviews [10] were conducted with all ten participants to understand their experiences of living with severe PF injury. The questions were neutrally phrased and designed to elicit personal insights, based on the study’s research questions, prior observations, and literature review. The format allowed participants to speak freely, covering topics such as the impact of PF injury on daily life, interaction with healthcare before, during, and after pregnancy, and their hopes, fears and advice for others and healthcare professionals.

### The Qualitative Content Analysis

Qualitative content analysis (QCA) was used to analyse interview data, focusing on participant experiences and context [12–14]. Following Elo and Kyngäs [15] and Sandelowski [16], an inductive approach was applied, allowing categories to emerge from the data rather than from theory. This method supported the discovery of unanticipated insights and was well-suited for exploring the lived experiences of PF injury. However, it required careful execution and transparent reporting. Interview results were analysed using QCA based on Graneheim et al.’s framework [12], following these steps:

1. The research team jointly defined the aim of the analysis.
2. The condensed transcripts (created by the first author) were imported into NVIVO [17] for review and coding by the team to start analysis.
3. Codes were compared to identify patterns, forming clusters and categories through iterative discussions and transcript reviews.
4. Similar codes were grouped into subcategories and categories, with reliability checked and minor adjustments made collaboratively.
5. Themes were developed from the categories to capture the deeper meaning behind women’s experiences of PF injury and healthcare interactions.

#### Comics as a Mode of analysis

Comics-based research refers to an emerging methodological development of using comics within the research process [6]. Creating comics during QCA helped capture key narratives and deconstruct the complexities of the PF injury journey. Comics provided multimodality through sequence, visuals, humour, and text, which can help alleviate the burden of heavy subjects related to PF care and offer new perspectives. In this study, comics were not used during data collection. Instead, they emerged parallel to the QCA as a way to synthesise and interpret the findings from a critical perspective using satire and the UD framework, and to support the visual dissemination of the results. The comics method in this study was co-created with guidance from Robert Nyberg, a professional comic artist in Sweden [18].

### Methodological Discussion

Although the methodology was reported transparently, its trustworthiness was influenced by the limited number of participants, consisting of ten women. As such, the findings were context-specific and did not aim to represent all aspects of the phenomenon. On the other hand, ten interviews provided valuable insights and were considered sufficient to reach saturation within this context. Newborns’ presence in some interviews may have influenced the interview situation. However, it has contributed to understanding the transformative period women were going through. This aspect was therefore considered part of the natural setting of the interviews.

During the QCA process, the definitions of subcategories were chosen to sort the manifest content, while underlying meanings informed the development of subthemes and overarching themes. The theme is described as a “unifying red thread” that becomes apparent once identified [12]. Patterns emerged from the data-guided abstraction and interpretation, allowing the narratives to be meaningfully understood. Lindgren et al. [14] states that abstraction and interpretation are core to the analytic process, and trustworthiness is enhanced through detailed descriptions of the analytic process. According to Graneheim and Lundman [13] thorough documentation supports transferability. In this study, the QCA process and results were described in sufficient detail to clarify both strengths and limitations [15].

The multidisciplinary working group, comprised a registered physiotherapist with experience in women’s health and PhD, a graphic design lecturer, and a PhD student in design, brought different perspectives to the analysis, enhancing confirmability. Even so, there was a concordance in understanding the content, which could be a strength in the credibility of the analysis. As the primary instrument, the researchers’ awareness of their own preconceptions and influence on both data collection and analysis was analysed and monitored [12]. The first author conducted both the observations and interviews. Reflexively acknowledging this dual role was therefore important. As a researcher with a background in design, the first author may be more attentive to special arrangements, visual aspects, and interactions between people and objects within the room. This perspective may influence how observations and interviews are conducted and interpreted. Throughout the study, these potential influences and contributions with design sensitivities were discussed within the research team to support reflexivity and strengthen the credibility of the analysis.

Comics were used to represent sequences from women’s narratives, though choosing suitable depictions proved difficult. While emotionally impactful stories might appear engaging, care was taken to consider the emotional effect on participants, some of whom may have experienced trauma. A nuanced approach was employed to reflect the diversity of experiences among women with PF injuries. To safeguard participants’ identities, composite stories based on shared experiences were utilised. The QCA was conducted independently of the comics’ development to ensure that coding and themes are grounded in the textual data. The comics were created afterwards to synthesise and visually represent key situations identified in the analysis. They were made by the first author and are grounded in participants’ narratives to minimise the risk of oversimplification or aesthetic bias.

### Ethical Considerations

This study has received approval from the Swedish Ethical Review Authority prior to the study start. All participants were assigned coded identifiers to ensure anonymity.

## Results

**Fig. 1:**
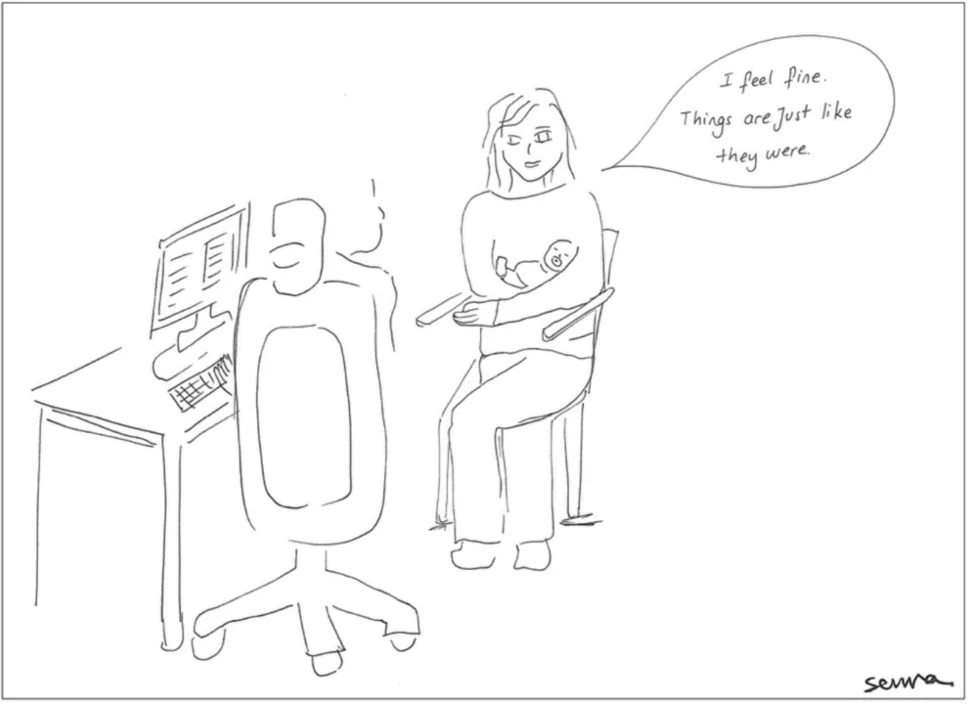
A woman with her newborn and a doctor in the consultation room. The woman says, “I feel fine; things are just like they were”

The Women’s PF injury narratives ranged from “everything is just as before” to “fear of leaving the house”. Emotions such as sadness, shame, stress, fear and worry appeared in stories about tears and related issues. Stress, worry, and fear were tied to childbirth and hospital experiences, while shame often emerged later, linked to incontinence during the return to daily life. Some participants expressed positive feelings, especially those with fewer symptoms, believing in recovery. See Fig. 1 for a comic illustration of a participant who had no symptoms left after birth, contributing to the diversity in the narratives.

The QCA analysis highlighted two main themes. The first was Bubbleverse, a metaphor for women’s journey through birth-related bubbles. These included reflections on the birth experience, the way newborn care can overshadow PF injury, and their anticipations about the future. The second theme centred on healthcare recommendations based on the women’s perspectives. It emphasised the importance of timely, personalised communication and the need for preventive, structured support tailored to individual needs. Together, these themes offered insights into PF injury experiences and the healthcare received, pointing to ways services can better support affected women. See Table 2 for an overview of the themes and the analysis process. Refer to Table 3 for an example of the analytical pathway during the QCA process.

**Table 2:** Overview of the themes and the analysis process

	Theme 1	Theme 2
	The Bubbleverse – Women’s journey through bubbles	Recommendations to healthcare professionals
10 interviews ⇨ 100 codes ⇨ 7 categories	Subtheme 1⇧	Subtheme 1⇧
	The Birth bubble—Ongoing processing of the birth experience	Address matters as they arise
	Subtheme 2	Subtheme 2
	The Baby bubble—The newborn as the primary focus	Prospective care planning
	Subtheme 3	
	A Bubble on the horizon

**Table 3:** The QCA analytic pathway example

Subtheme	Category	Codes	Example quotation
Subtheme 1: The Birth Bubble – Ongoing processing of the birth experience	Feelings, reflections and coping	“Experiences of PF injury during birth”; “Partner received information”; “Communication and miscommunication during birth”; “Bubble, unclear memories of childbirth”	“I was so caught up in my own bubble with the pain… If no one had told me, I wouldn’t have known”. (Participant 4) “My partner heard what the midwife said, but I was in my own bubble and focused on the pain”. (Participant 9)
Subtheme 2: The Baby Bubble – The newborn as the primary focus	Family and support	“Baby prioritised over self”; “Limited awareness”; “Overwhelmed”	“I was completely out of it… I hardly cared – I had my baby with me”. (Participant 2)
Subtheme 3: A Bubble on the Horizon	Future perspective	“Hope for recovery”; “Uncertainty about future”; “Fear of complications”	“I hope that things continue to go well… Sometimes I think about it, but I believe I will recover”. (Participant 8)

Theme: The Bubbleverse—Women’s journey through bubbles during and after birth

### Theme 1. The Bubbleverse—Women’s Journey Through Bubbles

PF injuries often receive less attention, overshadowed by the joy of holding the baby or other complications. Though unexpected, injuries were not immediately processed as the focus lies elsewhere. Reflections typically emerged weeks later. Birth involves a complex transition from pregnancy to motherhood. This was visualised through evolving, interconnected *bubbles*, each phase (during pregnancy, birth and early weeks after birth) linked to the next—forming the Bubbleverse.

The metaphor of the Bubbleverse illustrates how women’s attention and experiences shift through interconnected phases of childbirth and early recovery, helping express aspects of the postpartum experience that are otherwise hard to articulate. From a design perspective, the value of such a metaphor lies not in itself, but in its use. To discuss prospective futures, bubbles afford a way of thinking, a metaphor, that can potentially serve as an organising principle for this period, helping to anticipate what lies ahead.

#### Subtheme 1. The Birth Bubble—Ongoing Processing of the Birth Experience

Women’s memories and emotions surrounding childbirth varied. Some recalled events clearly, while others retreated into a bubble due to pain, medication, or stress—sometimes leading to isolation and disconnection. A sense of lost control was common. Reflections surfaced weeks later, often questioning what could have been done differently. From a UD perspective, this highlights the need for more responsive care practices that better address women’s needs during and after childbirth.

#### Partners as a Key Support

Partners played a vital role during childbirth and early postpartum, aiding communication and emotional processing. They offered support with PF injury care and were often the closest confidant. While some women mentioned family or friends, partners were most frequently cited.

**Fig. 2:**
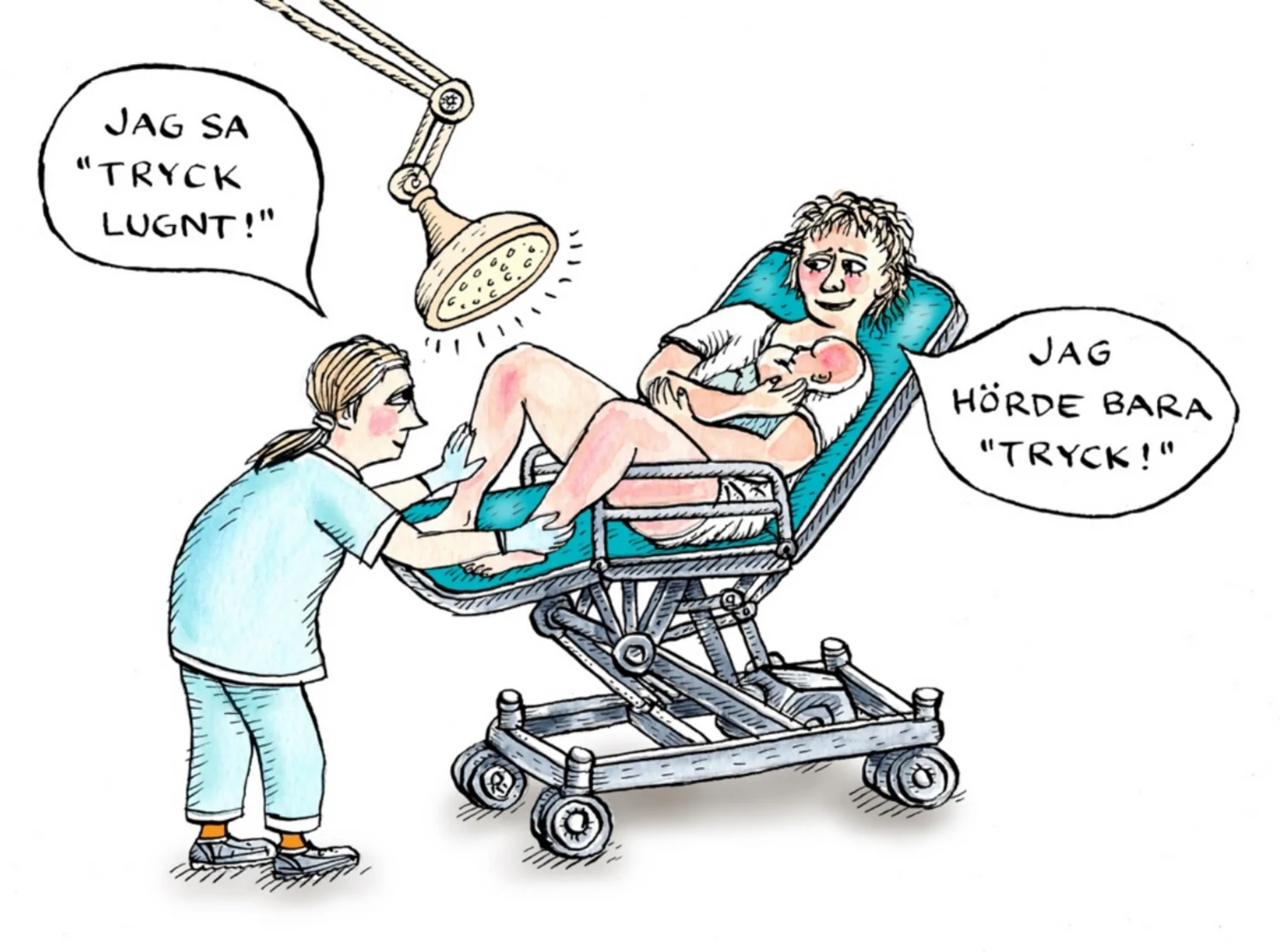
A comic illustration in which a midwife examines a woman’s PF and says, “I told you to push gently!” The woman holding her newborn baby in her arms replies, “I only heard push!”. ©illustration by Robert Nyberg, composition and idea development by the first author

Unpredictable events like sudden water breaking or rapid birth—appeared in many stories. Most women felt positively about midwife communication and care, though pain and stress during pushing made communication difficult. Many didn’t hear instructions clearly, recalling only “Push!” instead of “Push gently”. Figure 2 illustrates this miscommunication, often realised later through partner conversations describing what happened during birth.

#### Subtheme 2. The Baby Bubble—The Newborn as the Primary Focus

The first weeks after childbirth were crucial, though experiences varied. Joy over a healthy baby, becoming a mother, caring for the newborn, and building daily routines were central themes. Women focused intensely on the baby’s needs, forming *a Baby Bubble*—a known phenomenon. Many remained fragile and needed support on their terms. Their stories showed the newborn takes priority, with PF injury care adjusted accordingly.

**Fig. 3:**
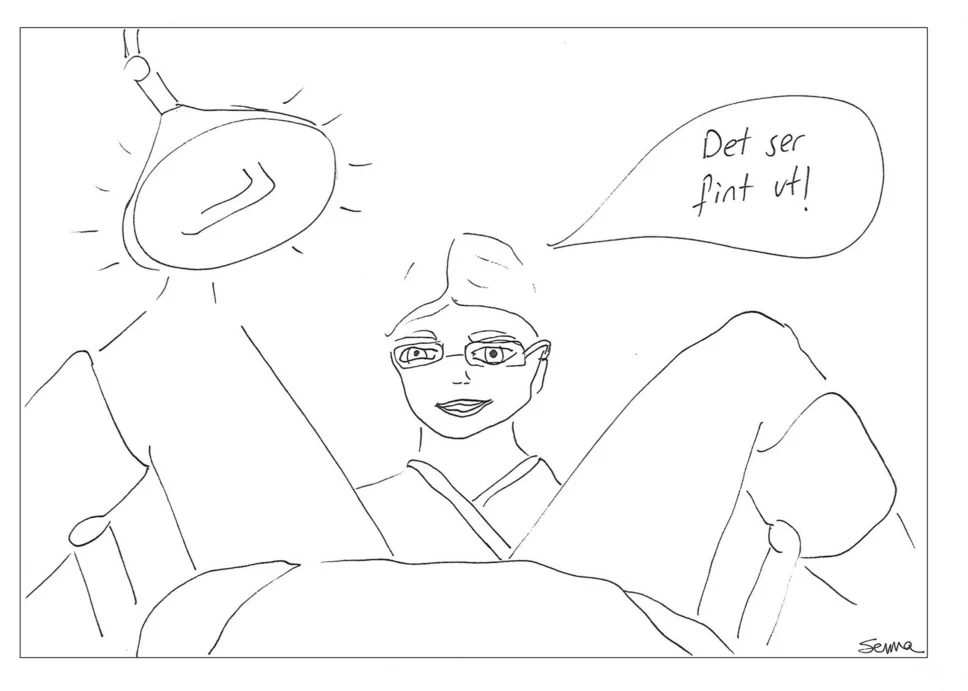
A comic illustration from a gynaecological examination room from the participant’s perspective. The participant’s legs are wide open, and the doctor is between the legs, saying, “It looks fine”. ©illustration, composition and idea development by the first author

The Standard check-up occurred during the Baby Bubble. The comic “It Looks Fine” in Fig. 3, illustrates women’s views during gynaecological exams. Some felt reassured by the phrase, while others felt dismissed—it may look fine, but did not feel fine. The comic aimed to spark dialogue about what “It looks fine” meant for those with PF injuries.

The baby remained a top priority even as daily life resumes. Women described the importance of their partner staying home in the early weeks and the loneliness when they returned to work. Pain during movement, rushing to the toilet while breastfeeding, and lifting the baby were common challenges. Toilet access, stool consistency, and laxatives were crucial early on—often purchased by the partner. Some women attempted walks but face chafing, pain, and incontinence. While some stayed active with little discomfort, others remained close to home, carrying laxatives, extra clothes, and food when going out.

#### Subtheme 3. A Bubble on the Horizon

The women expressed a strong desire for a functioning daily life, to begin PF exercises and training. Some reflected on future pregnancies and worried about grade three and four tears. While some lived symptom-free and hoped it continues, others feared impacts on intimacy, work, studies, and travel. They didn’t want lingering issues to limit everyday life.

#### Allow Time for Recovery

**Fig. 4:**
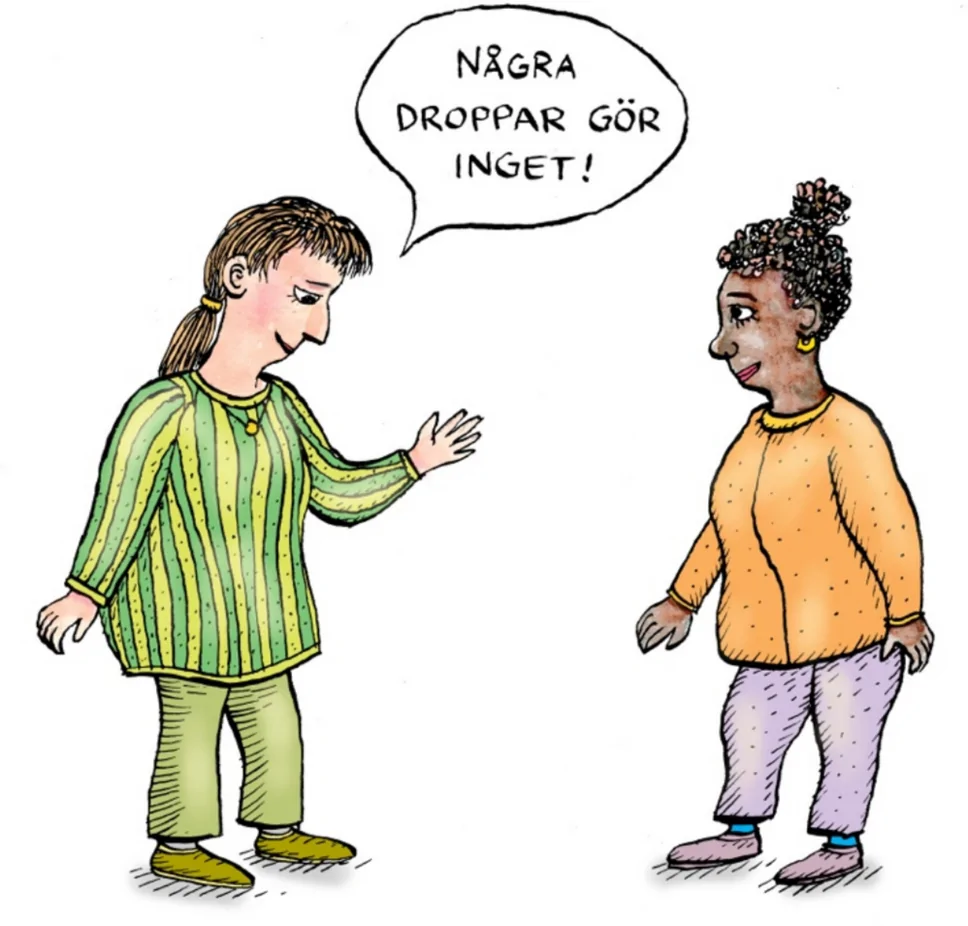
A comic illustration in which two women with urine leakage talk to each other, one saying “A few drops won’t hurt!”. ©illustration by Robert Nyberg, composition and idea development by the first author

“Give it time” was a common piece of advice among women with PF injuries. They had learned that healing takes time and varies individually. Many participants emphasised that discomfort shouldn’t be normalised or ignored—seeking help is crucial. The comic, see Fig. 4, A Few Drops Don’t Hurt, challenges the normalisation of issues like urine incontinence by making the problem visible and breaking the silence. Women in the comic smile to cope—not because it’s funny.

### Theme 2. Recommendations to Healthcare Professionals

Despite injuries, most women were satisfied with the care. They described safe but informal access to specialists—unscheduled visits and calls in the first weeks. Their birth plans often differed from reality, making it hard to manage the unpredictable.

General advice to healthcare: Inform about what is known, what has happened, what is not known, and what options are available for seeking help in the future. Be attentive and helpful to the woman’s story and wishes; they know their body. Be responsive to their needs regarding food, toileting, and laxatives, particularly during the first few days after childbirth. An example of this is the first toilet visit after childbirth, which is an important milestone for the participants. While it goes well for some, others need significantly more help with their diet and medications.

#### Subtheme 1. Address Matters as they Arise

Many preferred receiving PF injury information when it occurs, rather than in advance. Communication and timing were crucial, as women varied in when and how they wanted to be informed. From a UD perspective, this calls attention to patient-centred, flexible communication ways that can meet women’s diverse needs. Some absorbed details immediately, while others needed days or weeks. Providing essential information while delaying less urgent details, could reduce reliance on online sources. Experiences with predelivery tear information differed. Some actively sought it from friends or online, while others overlooked it during pregnancy or midwife visits.

Stitching after childbirth received little criticism, but the operation itself caused fear. Most women separated the injury from their care and felt generally safe. However, some were not mentally prepared for the surgery news, and being separated from the baby was deeply upsetting. The transition to surgery was smooth, but the absence of the baby caused loneliness and longing. Communication during childbirth often faltered owing to stress and pain. Partners’ perspectives helped women process events and gain insights days or weeks later. Many reflected on what could have been done differently, both in their healing and future pregnancies. Healthcare providers should consider having a conversation about “Why did I tear?”.

Post-operation information is generally well-received. Doctors often explained the injury using drawings and mirrors, asking if women wanted to see their genital area. This guided approach was valued more than self-examination at home. Some women imagined their injury as worse than it is, and visual explanations helped correct these perceptions, becoming a milestone in their recovery.

#### Subtheme 2. Prospective Care Planning

The final check-up was an important moment for women to assess healing and ask about resuming daily life. Most were pleased with the care and information provided. Some saw this visit as a milestone, indicating they were ready for activities such as swimming or sexual intercourse.

**Fig. 5:**
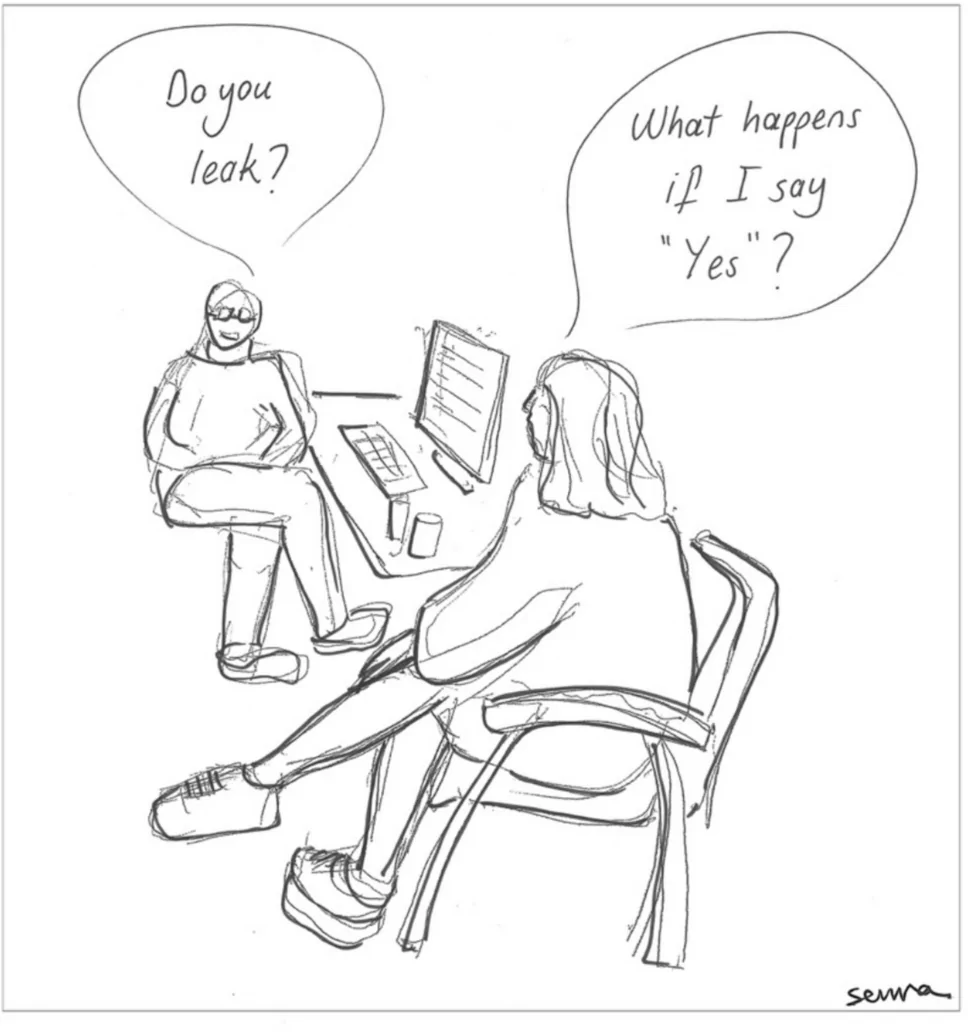
A comic illustration of a woman talking to a doctor in a consultation room. The doctor asks, “Do you leak?” and the woman answers, “What happens if I say yes?”. ©illustration, composition, and idea development by the first author

Future care varied: some receive a questionnaire a year after birth, others await a phone call. Women felt responsible for seeking help, expressed that support exists but must be actively pursued. From an accessibility perspective, this raises the need to structure preventive care plans. Concerns about long-term issues remained, and some hoped for proper care if problems persist. The comic presented at Fig. 5,*What happens if I say “yes”?* questions fragmented PF aftercare.

## Discussion

The first weeks after childbirth are critical, with women needing strong support—primarily from partners, but also healthcare. During this time, healthcare can ease the burden by organising postnatal care, sending reminders, and allowing women to stay focused on their baby bubble without needing to chase care.

A motivational conversation about PF injury a few weeks postpartum, using open-ended questions, can support emotional processing. Women are still trying to understand their injury, and some are considering future pregnancies. Timing is key—ideally, a few weeks after birth.

Support with the first bowel movement is a major milestone. Preventive advice and dietary guidance to ease this process should be further developed.

### Components of the Birth Bubble

Multimodal methods for communicating with the woman during the pushing phase are an area with development potential. Some women mentioned how they could hear their partner better, as he was physically positioned closer.

### Acknowledging the Baby Bubble

The period during which the participant is away from their baby and partner is an area that suggests opportunities for improvement. The participants wonder how the baby is doing; they get upset being away from their baby.

### Figurations of Aftercare

PF injury aftercare is poorly structured. Designing care that reflects the diverse healing journeys of women is a promising opportunity. UD methods can help facilitate flexible, inclusive care pathways. Women’s active participation in their own care should be supported, especially during this intense life phase. Healthcare can share responsibility by initiating contact and structuring follow-up, making care more adaptive and responsive.

Healthcare professionals also work within their own “care bubble”, while women are in their “baby bubble”. Recognising these parallel experiences may uncover ways to enhance care.

### Form of the Birth Plan Letter

Healthcare providers should discuss the scope of the birth plan letter further if it requires revision, ensuring women are aware of the unpredictability of birth.

### Reflection Mechanisms and Support Systems

PF injuries are often deprioritised, reflected in how women do—or don’t—process them. Some only began reflecting after being invited to this study or through conversations with their partner. Structured, nonintrusive ways to support reflection without disrupting the baby bubble are worth exploring.

Partners are often the only consistent support through pregnancy, birth, and postpartum, offering a unique perspective. However, not all women have a partner or support system. Design solutions must account for this range from strong support networks to none, to align with the UD framework.

### Sociocultural Aspects

Society often normalises post birth discomfort, especially around taboo topics such as urinary leakage (and, for some women, fecal leakage), which can make it difficult for women to seek help or discuss their symptoms openly. These normalising structures, which silence discomfort, need further study.

Language also matters. Even the native Swedish speakers found it hard to express their physical and emotional experiences. Post-birth changes make articulation difficult, and language or cultural barriers add further complexity. These must be addressed in designing better PF care journeys.

### Contributions and Future Research

Our findings align with existing qualitative and clinical literature showing that women often experience prolonged physical symptoms, emotional distress, and challenges navigating healthcare after obstetric anal sphincter injuries (OASIS). Previous studies have described women’s experiences of physical and psychological limitations, uncertainty about recovery, and fragmented support in the postpartum period. For example, women with OASIS have reported extensive pain, crushed expectations about recovery, and difficulties in accessing appropriate care in the first months after injury [19]. Similarly, qualitative work has documented ongoing struggles with bodily changes and recovery up to 1 year postpartum, including impacts on body image and daily life [20]. A Swedish interview study also emphasized the need to individualize rehabilitation and communication, echoing our participants’ calls for tailored support [21].

Our study extends this literature by conceptualising women’s recovery across interconnected phases (The Bubbleverse) and by highlighting actionable recommendations for structured, individualized communication and follow-up. While the use of comics offers an innovative way to represent participants’ experiences visually, the core contribution lies in the depth of insight into how women perceive and sequence their postpartum journeys, and how care can be reconfigured to better meet their needs.

This article emphasises the complexity of the experience of receiving and living with an extensive PF injury through the Bubbleverse metaphor and the use of comics as an expression of the women’s perspectives. Discussing the implications of bubbles may afford ways to make them more noticeable and recognisable. Through satirical comics and metaphors such as bubbles, this study aspires to contribute to a further developed discourse and new questions that could yield clearer answers for improving women’s care. It underscores the necessity for care practices that are both contextually situated and inclusive. Future research should prioritise collaborative methodologies, incorporating women, their partners, and healthcare professionals in the development of more inclusive healthcare systems.

## Data Availability

Data availability is restricted because of ethical considerations and participant confidentiality requirements. The data are therefore not publicly available.
